# Development of Next Generation Stevia Sweetener: Rebaudioside M

**DOI:** 10.3390/foods3010162

**Published:** 2014-02-27

**Authors:** Indra Prakash, Avetik Markosyan, Cynthia Bunders

**Affiliations:** 1 The Coca-Cola Company, Atlanta, GA 30313, USA; E-Mail: cbunders@coca-cola.com; 2 PureCircle Limited, Lengkuk Teknologi, 71760 Bandar Enstek, Negeri Sembilan, Malaysia; E-Mail: avetik@purecircle.com

**Keywords:** specifications, rebaudioside M, rebaudioside A, purification, stability, food application, sweetener blends

## Abstract

This work aims to review and showcase the unique properties of rebaudioside M as a natural non-caloric potential sweetener in food and beverage products. To determine the potential of rebaudioside M, isolated from *Stevia rebaudiana* Bertoni, as a high potency sweetener, we examined it with the Beidler Model. This model estimated that rebaudioside M is 200–350 times more potent than sucrose. Numerous sensory evaluations of rebaudioside M’s taste attributes illustrated that this steviol glycoside possesses a clean, sweet taste with a slightly bitter or licorice aftertaste. The major reaction pathways in aqueous solutions (pH 2–8) for rebaudioside M are similar to rebaudioside A. Herein we demonstrate that rebaudioside M could be of great interest to the global food industry because it is well-suited for blending and is functional in a wide variety of food and beverage products.

## 1. Introduction

High-purity rebaudioside M (also known as rebaudioside X), is a natural non-calorie sweetener being commercialized jointly by PureCircle Limited and The Coca-Cola Company for food and beverage use. Rebaudioside M is one of the minor sweet components of *Stevia rebaudiana* Bertoni, a South American plant. It is a glycoside of the *ent*-kaurene diterpenoid aglycone known as steviol, and is found in nature accompanied by at least ten other sweet-tasting steviol glycosides [1,2,3]. Stevia sweeteners have been approved for use as a sweetener in a number of countries, including US, EU, Japan, China, Brazil and other countries [4].

### Discovery of Rebaudioside M

**Figure 1:**
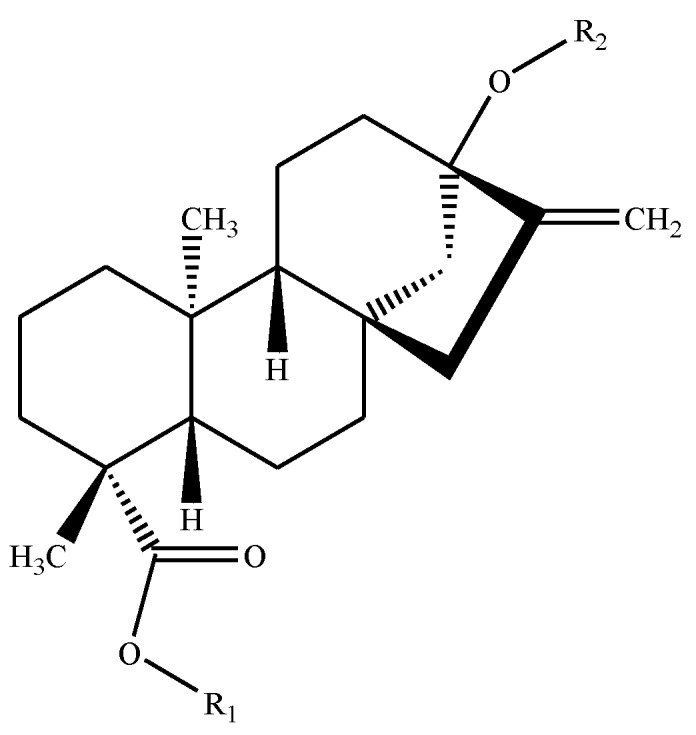
Backbone figure of *Stevia* sweeteners.

The leaves of *Stevia rebaudiana* Bertoni have been used by the natives of Paraguay to sweeten beverages for centuries [5]. The plant is the source of a number of sweet *ent*-kaurene diterpenoid glycosides (Figure 1, Table 1), but the major sweet constituents are rebaudioside A (**1**) and stevioside (**8**).

**Table 1:** R-groups, molecular formulas, molecular weights and potencies of the *Stevia* sweeteners.

Sweetener	Reference Number in Text	R-Groups in Backbone Figure Above		Formula	Molecular Weight (g/mol)	Potency *
		R_1_	R_2_			
Rebaudioside A	**1**	β-glc-	(β-glc)_2_-β-glc-	C_44_H_70_O_23_	967.01	200
Rebaudioside B	**2**	H	(β-glc)_2_-β-glc-	C_38_H_60_O_18_	804.88	150
Rebaudioside C	**3**	β-glc-	(β-glc, α-rha-)-β-glc-	C_44_H_70_O_22_	951.01	30
Rebaudioside D	**4**	β-glc-β-glc-	(β-glc)_2_-β-glc-	C_50_H_80_O_28_	1129.15	221
Rebaudioside E	**5**	β-glc-β-glc-	β-glc-β-glc-	C_44_H_70_O_23_	967.01	174
Rebaudioside F	**6**	β-glc-	(β-glc, β-xyl)-β-glc-	C_43_H_68_O_22_	936.99	200
Rebaudioside M	**7**	(β-glc)_2_-β-glc-	(β-glc)_2_-β-glc-	C_56_H_90_O_33_	1291.3	250
Stevioside	**8**	β-glc-	β-glc-β-glc-	C_38_H_60_O_18_	804.88	210
Steviolbioside	**9**	H	β-glc-β-glc-	C_32_H_50_O_13_	642.73	90
Rubusoside	**10**	β-glc-	β-glc-	C_32_H_50_O_13_	642.73	114
Dulcoside A	**11**	β-glc-	α-rha-β-glc-	C_38_H_60_O_17_	788.87	30

glc = glucose; rha = rhamnose; xyl = xylose; * Potency from [1,6,7].

In our continuing research to discover a natural non-caloric sweetener, we have focused on minor steviol glycosides and found a novel minor steviol glycoside, rebaudioside M (**7**) that is more potent, has higher sweetness intensity, and very slight licorice or bitter aftertaste than other steviol glycosides.

**Figure 2:**
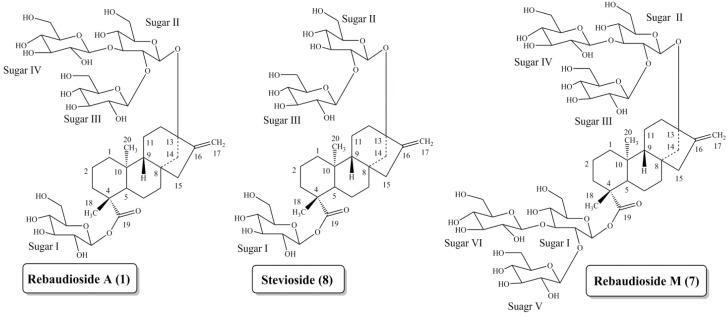
Comparison of the chemical structures of rebaudioside A (**1**), stevioside (**8**) and rebaudioside M (**7**).

Work to elucidate the chemical structures of *S. rebaudiana* sweeteners began in the early twentieth century, but proceeded slowly. The structures **1** and **8** (Figure 2) were not fully determined until 1970 [8,9,10]. During the 1970s, additional sweet components, including rebaudiosides A–E, were isolated from *S. rebaudiana* leaves and characterized by Osamu Tanaka and co-workers at Hiroshima University in Japan [11]. Several novel steviol glycosides have been reported from the commercial extracts of the leaves of *S. rebaudiana* in the last few years [6,12,13,14,15,16,17,18,19]. Recently we reported the structure elucidation and isolation of rebaudioside M from *S. rebaudiana* Bertoni (**7**) [20,21]. In addition, rebaudioside M received a Letter of No Objection concerning its Generally Recognized as Safe (GRAS) status from US FDA [22].

## 2. Experimental Section

### 2.1. Purification of Rebaudioside M

Rebaudioside M (**7**) was obtained from *Stevia rebaudiana* Bertoni cultivar AKH L1 leaves [23]. Two kilograms of *S. rebaudiana* leaves were extracted by 40 L water at 40 °C for two hours. The filtrate was separated and treated with a flocculant (calcium oxide) to remove the mechanical particles, proteins, polysaccharides and coloring agents. The resulting precipitate was separated by filtration and the filtrate was deionized by Amberlite FCP22 (H^+^) and Amberlite FPA53 (OH^−^) ion-exchange resins. The deionized filtrate was fractionated by seven columns, packed with Diaion HP20 (Mitsubishi Chemical, Japan). The columns with higher rebaudioside M content were eluted using aqueous ethanol. The obtained glycoside eluate was treated with activated carbon, deionized, evaporated and dried *in vacuo*. The obtained material was recrystallized twice from aqueous methanol to afford about 1.1 g of rebaudioside M with >98% purity by HPLC [21]. The highly purified stevia extract meets the international Joint Expert Committee on Food Additives (JECFA) purity requirement for steviol glycosides, which requires ≥95% total steviol glycosides [7].

### 2.2. Properties of Rebaudioside M

Pure rebaudioside M (**7**) (>95%) crystallizes out as a crystalline form which is slightly soluble in water (0.1 g/100 mL at 25 °C in 5 min) and in ethanol. Its amorphous material has a solubility of 1.1%–1.3% in water at 25 °C. The thermodynamic equilibrium solubility in water is 0.26% at 25 °C [24].

### 2.3. Stability of Rebaudioside M

As a dry powder, rebaudioside M (**7**) is stable for at least one year at ambient temperature and under controlled humidity conditions. In solution, it is most stable in pH 4–8 and noticeably less stable below pH 2. As expected, the stability decreases with increasing temperature. Its stability is very similar to rebaudioside A [25]. 

**Figure 3:**
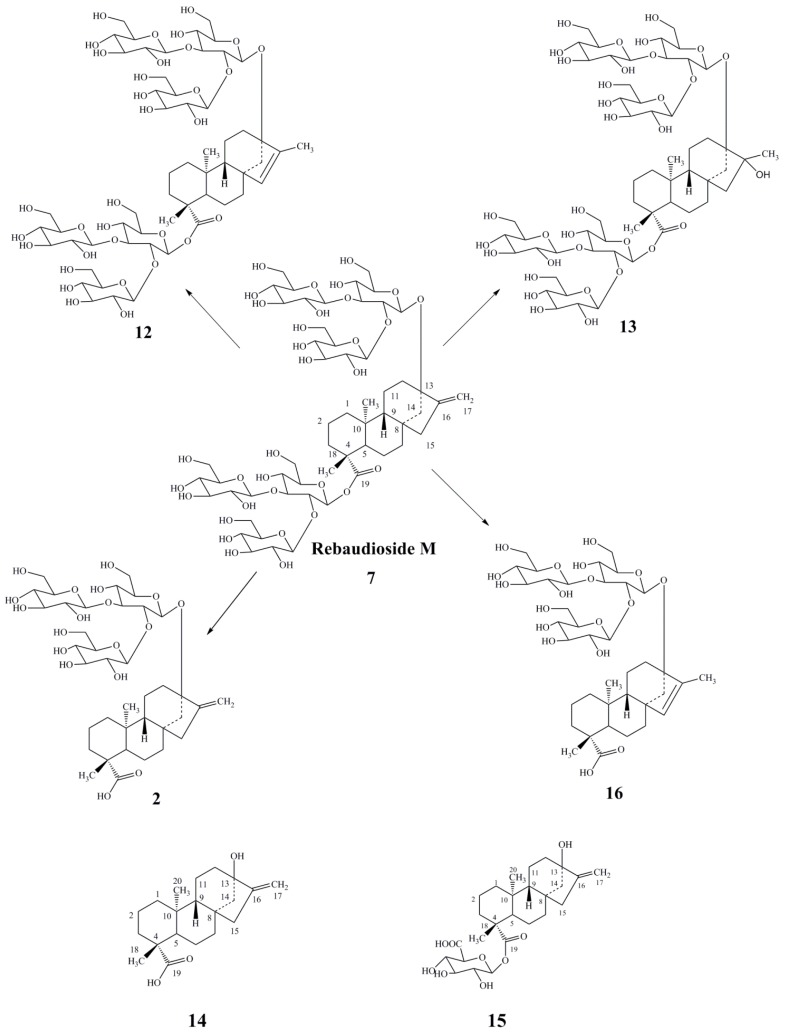
Major pathways of degradation of rebaudioside M (**7**) under hydrolytic conditions.

In aqueous solutions (pH 2–8), the major reaction pathways leading to loss of rebaudioside M are as follows (Figure 3): isomerization of the C-16 olefin to form the C-15 isomer (**12**), hydration of the C-16 olefin to yield compound **13**, hydrolysis of the glycosidyl ester at C-19 to form compound **2**, and isomerization of the C-16 olefin to form the C-15 isomer in **2** to form compound **16**. All of these compounds (**2**, **12**, **13** and **16**) are sweet. Compound **12** is sweeter than compounds **2**, **13** and **16**, and has similar taste properties as that of compound **7**. Upon metabolism *in vitro*, rebaudioside M (**7**), just like rebaudioside A (**1**), is primarily converted to its aglycone steviol (**14**) and to the glucuronic acid of steviol, generally known as steviol glucuronide (**15**) [26].

Rebaudioside M (7) has similar stability as that of rebaudioside A (1) in both low and high pH applications. In heat-processed beverages, such as flavored ice-tea, juices, sport drinks, flavored milk, drinking yogurt and non-acidified teas, the sweetener shows good stability during High Temperature-Short Time heat processing and on subsequent product storage [27].

### 2.4. Sensory Panel for Rebaudioside M and Other Sweeteners

To determine the sensory attributes of rebaudioside M (**7**) we compared it to rebaudioside A (**1**), which possesses well defined characteristics. In addition, blending techniques with rebaudioside M and other sweetener were examined to demonstration the potential of this sweetener. Herein we describe our sensory panel testing. Concentration-response (C/R) function work was done with 60 in-house trained panelists (Two-Alternative Forced Choice, 2-AFC method), with the panelists specifying which of the two samples was perceptually sweeter. Sweetener blend work was done with 8–10 descriptive analysis (DA) panelists that have been highly trained. DA panelists go through 10–12 weeks of extensive descriptive training. The panel was calibrated at the beginning of each test session. In addition the panel also received anchor references representing early (sucrose), middle (aspartame) and late (thaumatin) AT every three test samples. Samples were given to the panel members sequentially and coded with triple digit numbers. The order of sample presentation was randomized to avoid the order of presentation bias. Water and unsalted crackers were provided in order to cleanse the palate. The panel members were asked to rate different attributes including sweetness onset, total sweetness, rounded sweetness, bitterness, acidity, leafy note, licorice, astringency, mouthfeel, mouth coating, sweet lingering, and bitter lingering. Samples were rated on a scale of zero (0) to ten (10), with zero indicating immediate onset, no intensity, watery/low viscosity, or very sharp peak, and ten indicating very delayed onset, high intensity, thick/high viscosity, or very round peak. One-way single factor ANOVA was used to analyze sensory results, where α = 0.05.

## 3. Results and Discussion

### 3.1. Rebaudioside M Sensory Attributes

**Figure 4:**
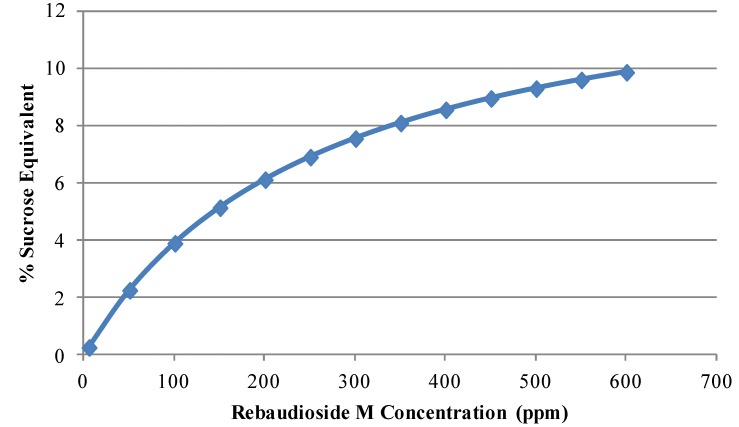
Concentration-response curves of rebaudioside M (**7**) in water at 4 °C.

To decipher the potential of rebaudioside M as a high potency sweetener, we first examined it against sucrose with the Beidler Model (Law of Mass Action) [28]. We used several reference samples of 2.5%, 5.0%, 7.5% and 10.0% sucrose solutions. Since sweetness potency is strongly dependent on sucrose equivalency level for all high potency sweeteners (HPS), it is important to state the sucrose equivalency (SE) level at which sweetness potency has been determined. Sweetness potency is also system dependent and therefore, it is important to define the medium (e.g., water, phosphoric acid at pH 2.5, *etc.*). The concentration-response (*C*/*R*) function in water determined for rebaudioside M is *R* = 14.2 × *C*/(265 + *C*) at 4 °C. Given the high *R*_m_ of 14.2, rebaudioside M can be used both in single and blended sweetener applications. From the *C*/*R* function the potency of water (P_w_) is P_w_(5) = 347 and P_w_(10) = 159 (Figure 4). This model estimates that the potency of rebaudioside M is 200–350 times that of sucrose.

**Figure 5:**
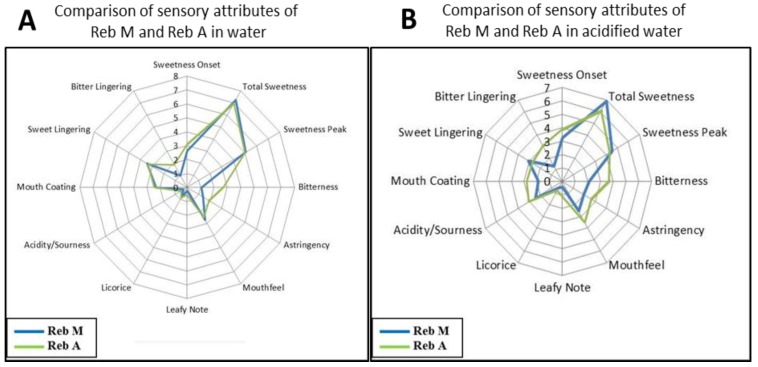
Comparison of sensory attributes of rebaudioside M (Reb M) (**7**) and rebaudioside A (Reb A) (**1**) in water (**A**). Comparison of sensory attributes of rebaudioside M and rebaudioside A in acidified water (**B**).

To enlist a broader picture of rebaudioside M (**7**) sensory attributes, we compared it to rebaudioside A (**1**). To compare the sensory attributes between rebaudioside M (**7**) and rebaudioside A (**1**), iso-sweet samples (8% sucrose equivalent sweetness) were made with filtered water as shown in Table 2. An 8% sugar solution in water was used as a control. The same concentrations of rebaudioside A and rebaudioside M were also used in acidified solutions (250 ppm citric acid, pH 3.2) and an 8% sugar solution acidified with citric acid was used as a standard. As described in Section 2.4, sensory panelist examined the two sweeteners and a spider plot was extracted (Figure 5).

In water solution, rebaudioside M (**7**) showed (Figure 5A) the reduced perception of bitterness, astringency, bitter lingering compared to rebaudioside A (**1**), and similar sweetness intensity. A much higher sweetness perception of rebaudioside M than rebaudioside A in acidified water was established in Figure 5B. Rebaudioside M displayed faster sweetness onset, reduced non-sweet taste (bitterness, sour, astringency) and bitterness lingering.

**Table 2:** Iso-sweet solutions of rebaudioside A (**1**) and rebaudioside M (**7**) in water.

Solutions	(%)	(%)
Water	99.95	99.95
Rebaudioside A 97 (dry basis)	0.0510 g	
Rebaudioside M (dry basis)		0.0423 g

**Figure 6:**
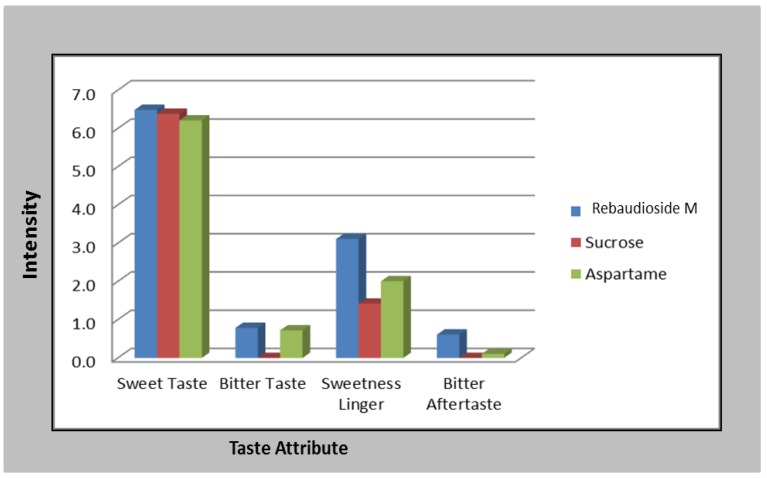
Descriptive taste profile of rebaudioside M (**7**) at 563 mg/L, aspartame at 531 mg/L and sucrose at 10% in water.

To expand the descriptive taste profile of rebaudioside M (**7**) a set of trained panelist (Section 2.4) examined 10% sucrose, aspartame at 531 mg/L, and rebaudioside M at 563 mg/L in water at 4 °C. Unlike other steviol glycosides including rebaudioside A (**1**), trained descriptive panel did not detect any significant bitter or licorice off taste when evaluated rebaudioside M in water at approximately 10% SE levels (Figure 6). Rebaudioside M had a slightly more intense sweet aftertaste then aspartame. Based on our study rebaudioside M and aspartame have similar high potency sweetener profiles.

Sweetness temporal profiles demonstrate changes in perception of sweetness over time. This property is a key to a sweetener’s utility in foods and beverages, and is complementary to its flavor profile. Every sweetener exhibits a characteristic Appearance Time (AT) and Extinction Time (ET). Most high-potency sweeteners, in contrast to carbohydrate sweeteners, display prolonged ET. This can be beneficial in some products such as chewing gum, where prolonged sweetness is desirable. 

With these samples in hand panelist examined the AT and ET of the three sweeteners described in Figure 6. The sweetness temporal profiles of aspartame at 531 mg/L, rebaudioside M at 563 mg/L, and sucrose at 10% in water at room temperature were compared over a period of 3 min (samples were swallowed at 5 s). The AT maximum was the shortest for sucrose, slightly longer for aspartame and longest for rebaudioside M. The ET was longest for rebaudioside M, followed by aspartame and then sucrose.

### 3.2. Blending

Blending of certain sweeteners (nutritive as well as non-nutritive) is often found to result in sweetness synergy. Such blends are also generally advantaged by improvements in flavor and temporal profiles as well as cost reductions in sweetener system and, often, improvement in stability [29,30,31,32,33].

To study the interactions between rebaudioside M (**7**) and other natural ingredients, blends were made with rebaudioside A (**1**), rebaudioside D (**4**), rebaudioside B (**2**), and erythritol at various concentrations in acidified water and sensory evaluation were performed. 

**Figure 7:**
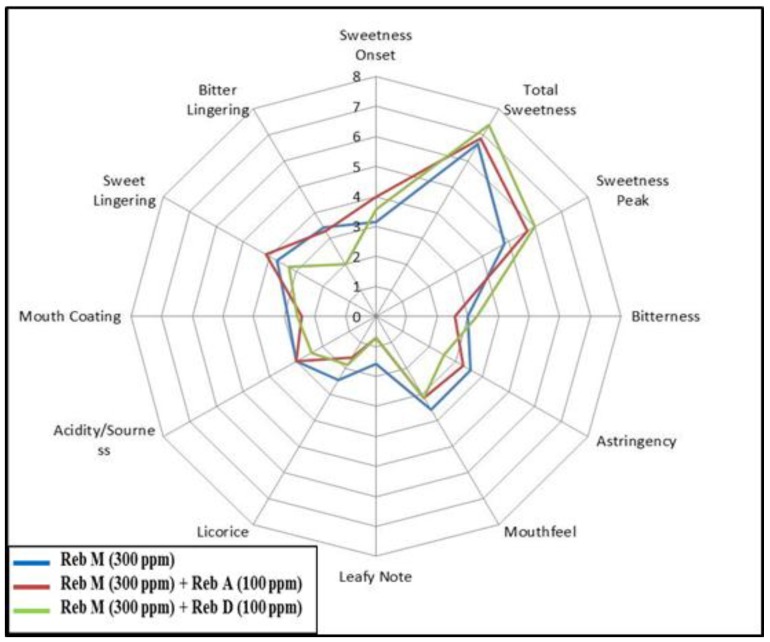
Sensory profile of di-blends of rebaudioside M (**7**) and rebaudioside A (**1**) or rebaudioside D (**4**) in acidified water.

Figure 7 illustrates the average response from sensory panelists that tasted the acidified solution with rebaudioside M (300 ppm) and rebaudioside A (100 ppm) or rebaudioside D (100 ppm) blends. The di-blends showed improvement in total sweetness, overall sweetness profile (peak), and leafy note. The blend with rebaudioside M and rebaudioside D showed higher improvement in sweetness intensity, overall sweetness profile, bitter lingering and sweet lingering.

**Figure 8:**
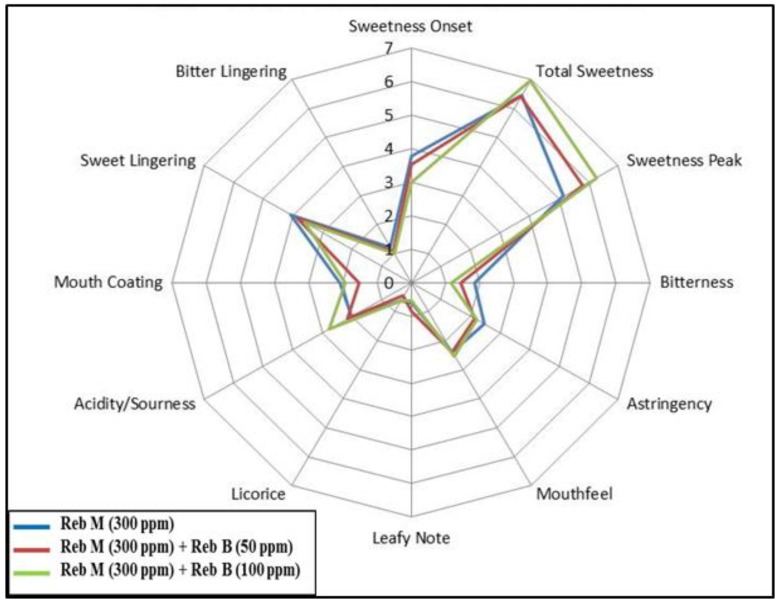
Sensory profile of di-blends of rebaudioside M (**7**) and rebaudioside B in acidified water.

The average response from sensory panelists that tasted the acidified solution with rebaudioside M and rebaudioside B blends (100 ppm or 50 ppm) is presented in Figure 8. The rebaudioside M and rebaudioside B di-blend exhibited additional rounded sweetness profile with slight improvement in sweetness intensity, onset and bitterness perception.

**Figure 9:**
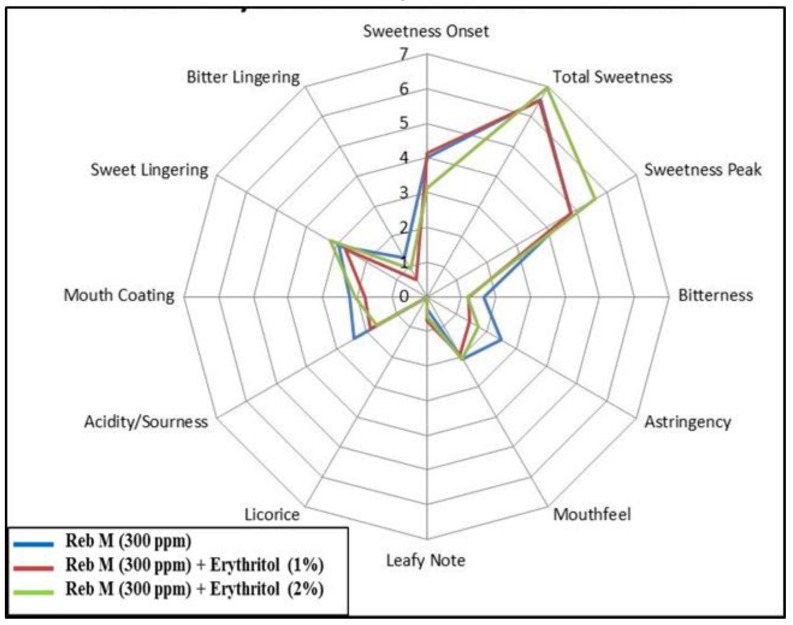
Sensory profile of di-blends of rebaudioside M (**7**) and erythritol in acidified water.

The taste description of rebaudioside M and erythritol is displayed in Figure 9 based on the average response from sensory panelists that tasted the acidified solution di-blend. The blend with erythritol helps in reducing acidity, bitterness, astringency and bitter lingering. At a higher level (above 1%, 100 ppm) erythritol contributes additional sweetness, rounded sweet profile and earlier onset.

Rebaudioside M (**7**) can be used as a single sweetener system in sparkling beverages and commonly employed in blends with a number of sweeteners. A wide range of both non-caloric and caloric sweeteners are suitable partners for rebaudioside M. This work illustrates a few of the possibilities for di-blend non-caloric sweeteners and how they alter sensory attributes. In addition, a blend where rebaudioside M contributes 20%–80% of the sweetness, and sucrose provides the rest, exhibits flavor and temporal profiles very close to that of sugar (short AT). Such blends of natural sweeteners offer (i.e., rebaudioside M and sucrose) substantial energy reduction over sucrose alone. Sweet tasting amino acids such as glycine, alanine, glutamine, proline and serine as well as salts such as sodium chloride and potassium chloride also improve the taste of rebaudioside M [21]. These blends also permit the formulations of good-tasting, natural, blended sweetener systems with lower energy content than carbohydrate sweeteners alone.

### 3.3. Food Application

The functionality and stability of rebaudioside M (**7**) were demonstrated with a three-dimensional food matrix model representing the intended conditions of use in foods [34]. Based on experience with rebaudioside A (**1**), the key factors likely to affect rebaudioside M stability in product were considered to be product moisture, process temperature, and product pH.

Products comprised of carbonated beverages, still non-alcoholic beverages, table-top sweetener formulations, chewing gum and yogurt. All products were packed, stored (mostly at 25 °C and 60% relative humidity) and evaluated at intervals using both chemical (HPLC) and sensory analyses. 

Sweetness of these products was assessed using panels consisting of 35 to 50 persons. Samples were evaluated using a five-point scale of categories ranging from 5 (much too sweet) to 1 (not at all sweet). Samples were considered satisfactory if at least 80% of the panelists rated the sweetness in category 3 (just about right) or above. 

In this study key findings were determined to provide insight of the sweetness properties of rebaudioside M (**7**) in a wide variety of products. In the category of soft drinks (both carbonated and still) cola and lemon-lime, sweetened with rebaudioside M, remained acceptably sweet throughout 26 weeks storage. For comparison, most soft drinks are consumed within 16 weeks of production. Concerning table-top sweetener, rebaudioside M was tested in a number of formulations and all were stable for at least 52 weeks. Rebaudioside M was considered stable and functional in chewing gum for 26 weeks. Plain yogurt was evaluated, it was deemed to have no significant loss of sweetness during measured pasteurization (190 °F for 5 min) and fermentation. Rebaudioside M was stable throughout a 6 week storage period (40 °F) in plain yogurt. Table 3 provides range concentrations of rebaudioside M for sweeten of various food and beverages.

**Table 3:** Typical rebaudioside M (**7**) concentrations used to sweeten various foods and beverages.

Product	Range ^a^ (mg/kg or mg/L)
Carbonated soft drinks	100–600
Still beverages	50–600
Powdered soft drinks (as is)	200–2000
Tabletop (as is)	800–4000
Bakery products	200–1000
Dairy products	150–1000
Chewing gum	300–6000
Confections	100–1000
Cereals	200–1000
Edible gels	200–1000
Nutraceuticals	200–1000
Pharmaceuticals	50–1000

^a^ Typical concentration when used as a single sweetener. Concentrations may vary depending upon formulation, flavor, and target consumer.

## 4. Conclusions

Through numerous evaluations we have determined that rebaudioside M has many beneficial properties and abundant potential as a sweetener in beverage and food products. This work illustrates that rebaudioside M has the capability to provide zero calories and has a clean sweet taste with slight bitter or licorice aftertaste. Functional in a wide array of beverages and foods, it is also well-suited for blending with other non-calorie or carbohydrate sweeteners. The molecule is stable under dry conditions and its stability is similar to rebaudioside A in aqueous food systems. High-purity zero-calorie natural stevia extract is of great interest to the global food industry because its natural source appeals to many consumers. 
